# Risk Prediction Models for Cardiotoxicity of Chemotherapy Among Patients With Breast Cancer: A Systematic Review

**DOI:** 10.1001/jamanetworkopen.2023.0569

**Published:** 2023-02-23

**Authors:** Elisé G. Kaboré, Conor Macdonald, Ahmed Kaboré, Romain Didier, Patrick Arveux, Nicolas Meda, Marie-Christine Boutron-Ruault, Charles Guenancia

**Affiliations:** 1 Health across Generations Team, Inserm U1018, Centre for Research in Epidemiology and Population Health, Villejuif, France; 2 Université Joseph Ki-Zerbo, Ouagadougou, Burkina Faso; 3 Department of Cardiology, CHU Dijon-Bourgogne, Dijon, France; 4 Center for Primary Care and Public Health, Unisanté, University of Lausanne, Lausanne, Switzerland

## Abstract

**Importance:**

Cardiotoxicity is a serious adverse effect that can occur in women undergoing treatment for breast cancer. Identifying patients who will develop cardiotoxicity remains challenging.

**Objective:**

To identify, describe, and evaluate all prognostic models developed to predict cardiotoxicity following treatment in women with breast cancer.

**Evidence Review:**

This systematic review searched the Medline, Embase, and Cochrane databases up to September 22, 2021, to include studies developing or validating a prediction model for cardiotoxicity in women with breast cancer. The Prediction Model Risk of Bias Assessment Tool (PROBAST) was used to assess both the risk of bias and the applicability of the prediction modeling studies. Transparency reporting was assessed with the Transparent Reporting of a Multivariable Prediction Model for Individual Prognosis or Diagnosis (TRIPOD) tool.

**Findings:**

After screening 590 publications, we identified 7 prognostic model studies for this review. Six were model development studies and 1 was an external validation study. Outcomes included occurrence of cardiac dysfunction (echocardiographic parameters), heart failure, and composite clinical outcomes. Model discrimination, measured by the area under receiver operating curves or *C* statistic, ranged from 0.70 (95% IC, 0.62-0.77) to 0.87 (95% IC, 0.77-0.96). The most common predictors identified in final prediction models included age, baseline left ventricular ejection fraction, hypertension, and diabetes. Four of the developed models were deemed to be at high risk of bias due to analysis concerns, particularly for sample size, handling of missing data, and not presenting appropriate performance statistics. None of the included studies examined the clinical utility of the developed model. All studies met more than 80% of the items in TRIPOD checklist.

**Conclusions and Relevance:**

In this systematic review of the 6 predictive models identified, only 1 had undergone external validation. Most of the studies were assessed as being at high overall risk of bias. Application of the reporting guidelines may help future research and improve the reproducibility and applicability of prediction models for cardiotoxicity following breast cancer treatment.

## Key Points

**Question:**

What is the evidence for use of clinical prediction models to predict cardiotoxicity related to chemotherapy treatment in women with breast cancer?

**Findings:**

This systematic review identifies 7 studies and 6 outcome prediction models for cardiotoxicity in different populations of patients with breast cancer. Most prognostic models report good to excellent discrimination but fall short in addressing bias due to methodological weaknesses, particularly for sample size, lack of overfitting consideration, handling of missing data, and insufficient model performance assessment.

**Meaning:**

These results suggest that there is insufficient evidence to provide personalized risk prediction of cardiotoxicity related to breast cancer treatment in clinical practice; thus, researchers should adhere to best practices in the process of developing and validating predictive models.

## Teaser

This systematic review evaluates risk prediction models of cardiotoxicity for patients with breast cancer.

## Introduction

Breast cancer is now the world’s most commonly diagnosed cancer, and it is responsible for 1 in 6 cancer deaths among women.^1^ Research has yielded several developments in breast cancer diagnosis and treatment that improve morbidity and mortality outcomes. Breast cancer survival 5 years after diagnosis now exceeds 80% in most high-income countries.^2^ Currently, there are more than 7.7 million breast cancer survivors around the world; this reflects improvements in diagnostics and treatment strategies including radiation therapy, chemotherapy, and biologic agents.^3,4^

Unfortunately, these therapeutics can cause potentially life-threatening adverse effects. Myocardial dysfunction and heart failure (HF), frequently described as cardiotoxicity, are serious adverse effects that women undergoing breast cancer treatment can experience.^5^ In the literature, the incidence of cardiotoxicity was reported to be as high as 34% when anthracycline and targeted agents are combined.^6^ Moreover, breast cancer survivors have a significantly increased risk of death due to cardiovascular disease (CVD).^7^

Given that cardiotoxicity is a significant factor in the prognosis for patients with breast cancer, many studies have aimed to identify predictors of cardiotoxicity. Age, obesity, hypertension, diabetes, and previous anthracycline treatment have been identified as major predictors of cardiac toxicity.^8,9,10,11^ Several scores that can assist health care professionals and patients with breast cancer in assessing prognostic indicators and other decisions made in primary care have been developed in this setting. With the increasing number of breast cancer survivors and adverse cardiovascular events, it appears important to systematically assess the available prediction models and their ability to predict cardiotoxicity. The purpose of this study was to identify, describe, and appraise all prognostic models developed to predict cardiotoxicity related to chemotherapy treatment in women with breast cancer.

## Methods

A systematic review was performed according to the Preferred Reporting Items for Systematic Reviews and Meta-Analysis (PRISMA) reporting guideline. The study protocol was registered with the Prospero International Prospective Register of Systematic Reviews (CRD42021290441). An ethics statement was not required because this study was based exclusively on published research.

### Inclusion Criteria for Study Selection

The PICOTS (population, intervention model, comparator, outcome, timing and settings) strategy was adopted for the search.^12^ We included all developed prognostic models and their corresponding external validation studies (intervention model), involving women diagnosed with breast cancer at any stage (population) who subsequently had cardiotoxicity, defined as changes in LVEF or all symptomatic cardiac toxic effects after breast cancer therapy (outcome), at any time during follow-up (timing), with no predefined comparator (comparator) or setting (setting). We excluded review articles, conference abstract and studies with only patients with metastatic breast cancer. Two reviewers (E.G.K. and A.K.) independently performed the first screening by title and abstract. The 2 investigators independently undertook all study processes including online database search, study selection, data extraction, and critical appraisal. Any disagreements were discussed to reach a consensus. When a consensus was not obtained, third-party experts were invited to research, discuss, and finally reach a decision.

### Search Methods for the Identification of Studies

On September 22, 2021, we searched the electronic bibliographic databases Embase, Medline (PubMed), and Cochrane with no restriction on publication date. We developed a search strategy from a combination of free text and Medical Subject Heading terms for BC, cardiotoxicity, and HF (eAppendix 1 in Supplement 1).

### Data Collection and Analysis

We adapted the standardized data extraction form containing items based on the general guidelines of the Critical Appraisal and Data Extraction for Systematic Reviews of Prediction Modelling Studies (CHARMS) checklist.^13^ Data were extracted for general information, source of data, participants, predictors, outcome, and analysis (eAppendix 2 in Supplement 1).

### Risk of Bias and Reporting Transparency Assessment

We used PROBAST (Prediction model Risk Of Bias Assessment Tool) to assess the risk of bias of the individual prognostic models investigated.^14^ Risk of bias (ROB) was assessed according to the 4 PROBAST domains (participants, predictors, outcome, and analysis) and applicability according to 3 domains (participants, predictors, and outcome). The ROB was assessed to be either high, low, or unclear based on signaling questions (eAppendix 3 in Supplement 1).

We used the Transparent Reporting of a Multivariable Prediction Model for Individual Prognosis or Diagnosis (TRIPOD) reporting guideline to assess good reporting of studies developing or validating multivariable prediction models (eAppendix 4 in Supplement 1).^15^ In the absence of sufficient data for a meta-analysis, we have used a narrative synthesis instead.

## Results

**Figure 1.**
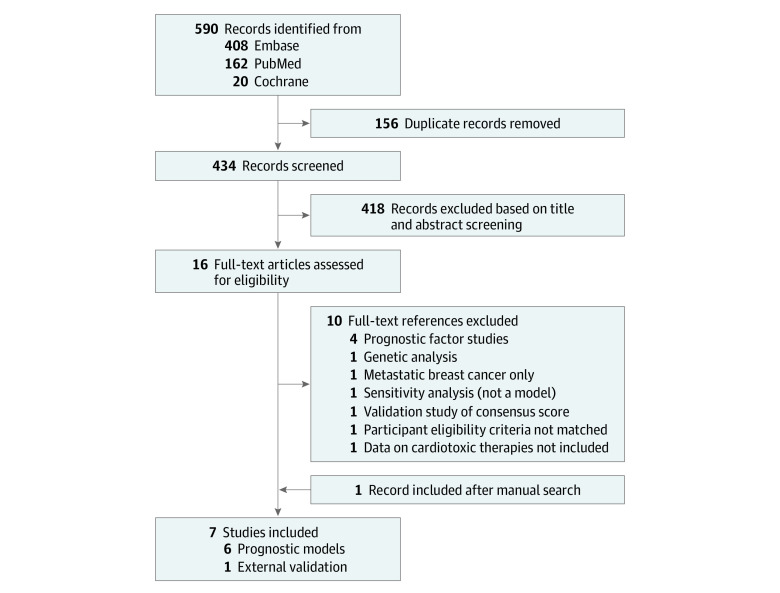
Flowchart of Study Inclusion

The search identified 590 potentially relevant citations related to prognostic models for cardiotoxicity in breast cancer. After removal of 156 duplicates, the titles and abstracts of the remaining 434 references were screened. We identified 16 studies for full-text assessment that would possibly fulfill our predefined inclusion criteria. Of these, we excluded 10 references (eTable 3 in Supplement 1). One study was included following a manual search.^16^ The PRISMA flowchart of the search strategy and the selection process is illustrated in Figure 1. Of the 7 studies included in this review, 6 focused on model development^16,17,18,19,20,21^ and 1^22^ focused on external validation (Tables 1 and 2).

**Table 1.** Model Development Studies With Their Predictors

Prediction model	Predictors (or risk factors) in the final model^a^	Outcome	Discrimination	Calibration
Ezaz et al,^17^ 2014; development model	Age 67-74 y, age 75-79 y, age 80-94 y, anthracycline chemotherapy, nonanthracycline chemotherapy, no identified chemotherapy, coronary artery disease, atrial fibrillation/flutter, diabetes, hypertension, and kidney failure	HF or CM defined as *ICD-9-CM* codes in ≥1 inpatient claim or 2 outpatient claims ≥30 d apart	Not reported	HL *P* = .76
Fogarassy et al,^18^ 2019; development model	Age 40-49 y, age 50-59 y, age 60-69 y, age ≥70 y, diabetes, hypertension, CAD without myocardial infarction or revascularization, CAD with myocardial infarction or revascularization, previous stroke, regional invasion, distant metastasis, epirubicin dose 451-540 mg/m^2^, dose 541-709 mg/m^2^, dose >709 mg/m^2^, docetaxel dose ≤510 mg/m^2^, docetaxel dose >510 mg/m^2^, capecitabine, gemcitabine, bevacizumab, ACEi/ARB	HF defined as (1) hospital discharge following the diagnosis of the *ICD* code I50, (2) hospitalization that ended in death and an I50 code issued as a primary or secondary diagnosis or as the underlying cause of death, (3) autopsy report with the I50 code	*C*-index, 0.79	HL *P* = .29
Low risk TRC; Goel et al,^19^ 2019; development model	Baseline LVEF and LVEF change^b^	Any of (1) death due to HF failure, AMI, or arrhythmia; (2) grade III or IV cardiac arrhythmia or ischemia or infarction; (3) NYHA III or IV; (4) an asymptomatic decrease in LVEF of >15%; and (5) an asymptomatic decrease in LVEF of >10% to an absolute value of <50%	*C*-index, 0.87 (95% IC, 0.77-0.96)	Not reported
CHEMO-RADIAT; Kim et al,^20^ 2021; development model	Prior congestive HF, Hypertension, age ≥60 y, prior myocardial infarction/peripheral artery occlusive disease, obesity, kidney failure, abnormal lipid profile, diabetes, radiation to left breast with ≥30 Gy dose, anthracycline dose, TIA/stroke	MACE: composite of HF, myocardial infarction, stroke, cardiovascular deaths	*C*-index, 0.87 (95% IC, 0.78-0.96)	HL *P* = .09
CRS; Romond et al,^16^ 2012; development model	Age and baseline LVEF	Definite or probable cardiac death or congestive HF manifested by dyspnea with normal activity or at rest and associated with an absolute decrease in LVEF of >10% from baseline to a value <55% or a decrease of >5% to a value below the lower limit of normal	*C*-index, 0.72	Plot between observed and predicted probability
Upshaw et al,^21^ 2019; development model	Age, BMI, hypertension, and baseline LVEF	Composite of reduction from baseline in LVEF ≥10% with a resultant LVEF <50% and/or a clinical diagnosis of heart failure through the first year of follow-up	*C*-index, 0.70 (95% IC, 0.62-0.77)	Not reported

Abbreviations: ACEi/ARB, angiotensin-converting enzyme inhibitors/angiotensin receptor blocker; AMI, acute myocardial infarction; BMI, body mass index (calculated as weight in kilograms divided by height in meters squared); CAD, coronary artery disease; CM, cardiomyopathy; GLS, global longitudinal strain; HF, heart failure; HL, Hosmer-Lemeshow; *ICD*, international classification of disease; LVEF, left ventricular ejection fraction; MACE, major adverse cardiovascular events; NYHA, New York Heart Association; TIA, transient ischemic attack.

^a^ Risk factors for Goel et al.^19^

^b^ Baseline LVEF is LVEF preanthracycline, and LVEF change is baseline LVEF minus postanthracycline LVEF.

**Table 2.** Study Characteristics of Model Development Studies

Source	Source of data	Participant characteristics				Outcomes		Presentation	Validation	
		No. of persons	Age, y	Stage	Treatment	Participants with cardiotoxicity, No. (%)	Follow-up		Development stage	External
Ezaz et al,^17^ 2014; development model	Retrospective cohort, US, registry data, January 1, 2000-December 31, 2009	1664	73.6 (5.3)	Stage 1: 26.8%; stage 2: 45.6%; stage 3: 27.6%	ANTH chemotherapy: 35.9%; non-ANTH chemotherapy: 47.7%; No identified chemotherapy: 16.4%	318 (19.1%)	3 y	Scoring system	Yes	Yes
Fogarassy et al,^18^ 2019; development model	Retrospective study, Hungary, registry data, January 1, 2004-December 31, 2016	8068	Not reported	Not reported (all stages)	100% Epirubicin-treated breast cancer; 20% of women with targeted therapies	557 (6.9%)	Median 5.89 (3-10) y	Scoring system	Yes	No
Low risk TRC; Goel et al,^19^ 2019; development model	Prospective cohort study, Australia, multicenter, recruitment period not reported	217	52 (28-77)	Not reported (early stage, *ERBB2* positive)	Conventional adjuvant ANTH-based chemotherapy followed by taxane chemotherapy given with TRZ, followed by TRZ alone to complete a total of 52 weeks	18	52 wks	Logistic regression	No	No
CHEMO-RADIAT; Kim et al,^20^ 2021; development model	Retrospective cohort, South Korea, multicenter, November 2005-September 2015	1256	51.4 (10.7)	Stage 0: 1%; Stage 1: 43.1%; stage 2: 38.5%; stage 3: 16.8%; Stage 4: 0.7%	Use of ANTH:70%; TRZ: 11.9%	21	48.7 mos (range, 25.8-71.8 mos)	Scoring system	Yes	No
CRS; Romond et al,^16^ 2012; development model	Prospective cohort, US, multicenter, February 21, 2000-April 29, 2005	1830	49.0	Not reported (histologically node-positive, HER-2 positive)	Arm 1: doxorubicin and cyclophosphamide for four cycles followed by paclitaxel for four cycles. Arm 2: same chemotherapy plus TRZ starting with the first dose of paclitaxel at a loading dose of 4 mg/kg followed by 2 mg/kg for 51 weeks.	37 of 944 patients vs 10 of 743 patients	87 mos	Logistic regression	Yes	No
Upshaw et al,^21^ 2019; development model	Prospective cohort, US, multicenter, November 2007-February 2011	967	51.5 (9.6)	Stage 1: 8%; stage 2: 36%; stage 3: 56%	Doxorubicin (cumulative dose, 240 mg/m2) and cyclophosphamide for 4 cycles (classical [every 3 weeks] or dose-dense [every 2 weeks] followed by 12 doses of weekly paclitaxel	51	47.6 mos	Logistic regression	Yes	No

Abbreviations: ANTH, anthracycline; TRZ, trastuzumab.

### Model Development Studies

Of the 6 model development studies, 3 produced scoring systems,^17,18,20^ while the other 3^16,19,21^ were logistic regression prediction models (eAppendix 4 and 5 in Supplement 1). The prognostic models were mostly developed after 2019.

#### Participants

Of 6 studies, 3 used retrospective data^17,18,20^ and 3 used prospective data.^16,19,21^ Three studies included patients with localized breast cancer,^16,17,21^ 1 study included patients with localized and metastatic breast cancer,^20^ and 2 studies did not report the cancer stage.^18,19^ The numbers of participants in the 6 studies ranged from 217 to 8068 patients, and the mean age ranged between 49 and 73 years. Anthracyclines, alone or combined with trastuzumab, were used in all women in 4 studies.^16,18,19,21^

#### Follow-up Duration

Follow-up ranged from 1 to 7 years. Two studies assessed the 1-year risk of cardiotoxicity.^19,21^ The other studies reported had a longer follow-up period: 3-year risk of HF or cardiomyopathy^17^; 3-to-10-year risk of HF^18^; risk of major adverse cardiovascular events (MACE) at 1, 3, and 7 years^20^; and 5-year risk of cardiac death or congestive HF.^16^

#### Outcome Definition

The definition of cardiac events was not uniform. Three of the included studies used clinical criteria^17,18,20^; 2 of them used administrative data with *International Classification of Diseases, Ninth Revision (ICD-9)* and *International Statistical Classification of Diseases and Related Health Problems, Tenth Revision (ICD-10)* codes to define HF,^17,18^ and 1 of them used MACE as the outcome, which was a composite of HF, myocardial infarction, stroke, cardiovascular deaths.^20^ In 3 studies, clinical criteria were used in conjunction with left ventricular parameters.^16,19,21^

#### Method, Analysis, and Presentation

The included models used between 2 and 20 predictors (Table 1). Most often used predictors were age^16,17,18,20,21^ and comorbidities. Comorbidities included: diabetes^17,18,20^; hypertension^17,18,20,21^; body mass index^20,21^; atrial fibrillation^17^; prior coronary artery disease^17,18,20^; kidney failure^17,20^; and prior stroke.^18,20^ Some studies explored other predictors such as: disease characteristics (regional or distant invasion)^18^; therapeutic methods used such as dose of chemotherapy agents (specifically anthracycline doses)^18,20^ or dose of irradiation to left breast^20^; biochemical predictors such as lipid profile^20^; and echocardiographic parameters such as baseline LVEF^16,19,21^ and LVEF change.^19^ The models and their calculations are described in eTables 4 and 5 in Supplement 1.

#### Performance

Calibration was reported in 4 studies.^16,17,18,20^ One study did not report discrimination.^17^ Of the 5 prognostic models for which the *C* index was reported, 2 models^19,20^ had excellent discrimination (0.80-0.89) and 3^16,18,21^ had acceptable discrimination (0.70-0.79), irrespective of cardiac event definition (Table 1).

#### Risk of Bias Assessment

**Figure 2.**
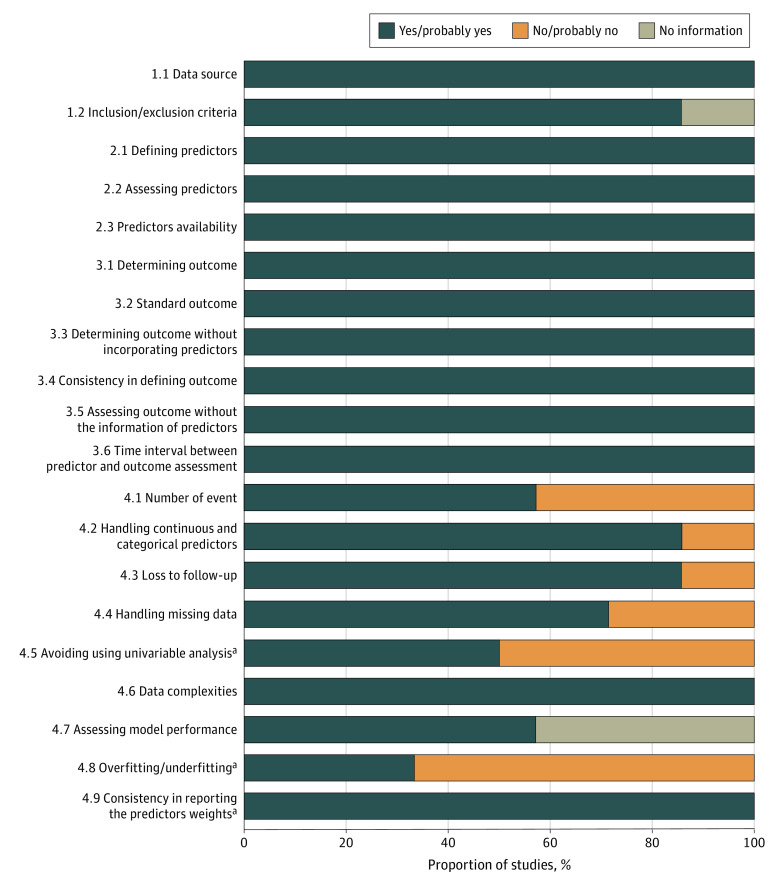
PROBAST Quality Analysis Results of 7 Studies Proportion of studies being answered Y/PY (yes/probably yes), N/PN (no/probably no), and NI (no information) for each PROBAST item. Development studies only.

In the PROBAST quality analysis, 1 study was rated as low ROB,^18^ 1 other as unclear ROB,^21^ and the remaining model development studies had high ROB due to concerns in the analysis domain (Figure 2; eFigure 1 in Supplement 1). Lack of overfitting consideration (item 4.8: internal validation techniques consists of random split-sample of participants^17,18,20^ or no internal validation has been performed^19^), reasonable number of participants with the outcome (item 4.1: fewer than 10 events per variable^19,20^), and participants lost to follow-up or with missing data handled inappropriately (item 4.3 and 4.4: participants with missing data^16,19^ or loss to follow up^19^ were excluded from analysis) were identified as bias related to analysis. We rated as low ROB the analysis domain for the prediction model of Fogarassy et al^18^ because the development study was based on a large data set and a high number of events per variable (EPV) stratum. In Upshaw and al,^21^ the development study was rated as unclear ROB due to insufficient model performance assessment (item 4.7: calibration not reported). We rated concern for applicability of all the development studies as low across all domains (eTable 6 in Supplement 1).

#### Transparent Reporting Assessment

**Figure 3.**
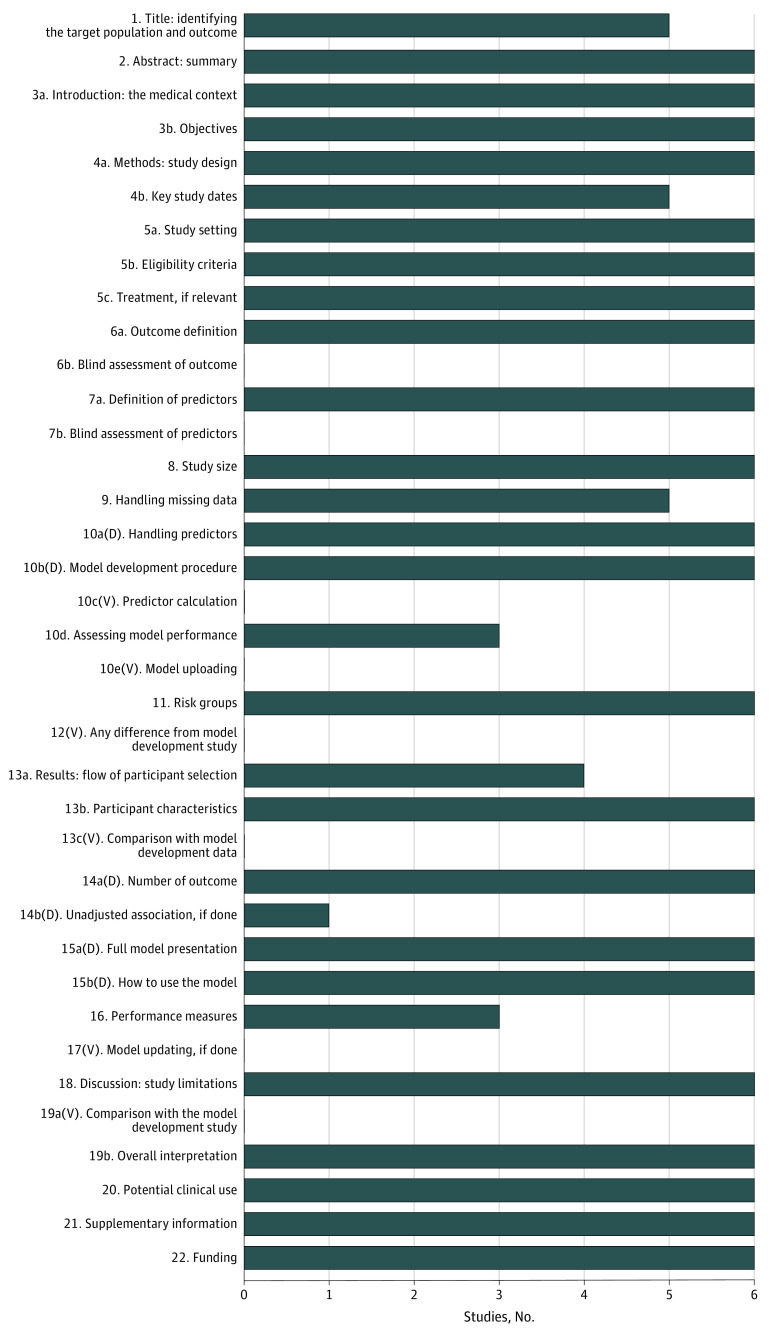
TRIPOD Checklist Numbers of development studies that reported each TRIPOD item. D, development study only; V, validation study only.

TRIPOD scores varied from 25 to 28 (of 31) in the 6 development studies (Figure 3; eFigure 2 in Supplement 1). All studies met more than 80% of the items in the TRIPOD checklist. Two studies^16,17^ were published before the TRIPOD’s publication in 2015. The items that were most often reported included the abstract (item 2), introduction (item 3), and source of data (item 4) for all studies. Lack of reporting on participant selection (item 13a) or models’ predictive performance (item 10d) were noted in 4 studies.^17,18,20,21^ Only 1 study^19^ reported unadjusted association between each candidate predictor and outcome (item 14b). No study reported masked assessment of outcome (item 6b) and predictors (item 7b). Missing data handling (item 9) was not reported in 1 study.^18^

### Model Validation Study

We identified 1 study (Milks et al^22^) externally validating the prediction model by Ezaz et al.^17^ It was derived from data from a retrospective, monocentric cohort study. The recruitment period for the study was from 2010 to 2014, and follow-up was not reported. Patients with all stage breast cancer treated by doxorubicin alone or in association with trastuzumab were included. A total of 183 patients were included with 33 events. The reported discrimination was excellent (AUC = 0.86). Calibration was not reported (item 4.7 rated “no information”). We rated the ROB as high due to analysis concerns, including item 4.1 (the number of participants with the outcome was under 100) and item 4.2 (categorical variables like age were categorized using cut-points that were different from those of the development study), and a lack of specified exclusion criteria (Figure 2). The TRIPOD score was 21 (total 31). Insufficient reporting of the title (item 1), source of data (item 4a), participants (item 5c, and 13), missing data handling (item 9), and model performance were identified.

In their study, Milks et al^22^ also suggest that superior prediction assessment for cancer-therapy–related cardiac dysfunction can be achieved by combining left ventricular global longitudinal strain (LV-GLS) and the clinical risk score from Ezaz et al.^17^ The combined model had superior receiver-operating characteristics (*C* = 0.9629).

### Presentation of Usability of the Models

Fogarassy et al^18^ was the only included study that had a low overall risk of bias. However, neither study was externally validated. None of the included studies explored net benefit analysis with respect to the developed models.

## Discussion

We conducted a systematic literature search that identified 7 studies and six outcome prediction models for cardiotoxicity in different breast cancer populations, including patients with breast cancer at any stage, *ERBB2* (formerly *HER2*)-negative patients, *ERBB2*-positive patients and patients eligible for trastuzumab. One of the models^17^ underwent external validation in a separate external validation study.^22^ The performance of these models and their applicability were assessed. Although most prognostic models reported good to excellent discrimination, of the 6 models, only 1 had low ROB and 1 other unclear ROB; the other 4 models had high ROB due to statistical analysis concerns, particularly for sample size, handling of missing data and not presenting appropriate performance statistics.

### Suggestions for Future Research

Two studies included in this review used registry data to develop their model. Registry data are commonly used in clinical prediction models because they provide large sample sizes and good representativeness while at the same time being relatively inexpensive and easy to obtain.^23^ Ezaz et al^17^ used the SEER-Medicare registry to identify 1664 American women diagnosed with early stage breast cancer, and Fogarassy et al^18^ used the Hungarian National Cancer Registry to include 8068 patients. Nevertheless, we should be cautious because the amount of detail in data collection and whether outcome assessment was conducted by protocol may be a limitation for prognostic analyses in patient registries.^24^

Two models included patients with advanced cancer stage (ie, metastasis),^18,20^ whereas other models included early breast cancer stage. As expected, in the study by Fogarassy et al,^18^ advanced cancer with distant metastases was confirmed as a significant predictor associated with heart failure. This likely reflects the choice of chemotherapy in addition to its potential impact on cardiotoxicity progression. Pinder et al^25^ published a large analysis of the Medicare database for patients with breast cancer older than 65 years, and identified advanced cancer stage as significant predictor for heart failure. In addition, Cho et al^26^ found that metastasis increased doxorubicin-induced cardiotoxicity by 2.66-fold and concomitant trastuzumab increased it by 4.08-fold. In the metastatic setting, *ERBB2*-targeted therapies are used until disease progression or toxicity.^27^ These findings suggest that cancer stage may be relatively important predictor in cardiotoxicity prediction models.

There was significant heterogeneity in the definition of cardiac events and follow-up. One study used a MACE outcome to develop a model to estimate the risk of MACE in women with breast cancer. Kim et al^18^ utilized a 4-point MACE outcome. Although MACE is an increasingly common and standardized primary outcome of interest in randomized controlled trials, there are potential limitations in studies using administrative databases. A systematic review reported substantial heterogeneity for the MACE composite endpoints used in studies based on administrative databases.^28^ “Acute myocardial infarction and stroke” and “acute myocardial infarction, stroke, and all-cause death” were the 2 most common composite MACE definitions. This diversity made it challenging to compare findings across studies or to aggregate multiple study results for meta-analyses or systematic reviews when considering different treatment or research questions.^28^

The diagnosis of cardiotoxicity is currently defined as a greater than 10% reduction in the ejection fraction (EF) and an absolute value of less than 53% according to the American Society of Echocardiography (ASE) and the European Society of Cardiovascular Imaging (EACVI),^29^ while the cutoff is 50% according to the European Society of Cardiology.^5^ In the past, the definition of cardiotoxicity related to chemotherapy was restricted to ventricular dysfunction. However, the introduction of new treatments in the last few years has widened the concept of cardiotoxicity to a broader definition of cardiovascular toxicity, which incorporates arterial hypertension, ischemia, cardiomyopathy, myocarditis, arrhythmic complications, long QT, and arterial and venous thrombosis.^30^ Future studies should therefore include a uniform and established definition of cardiotoxicity and a longer follow-up period to determine the effects of cancer treatments on long-term cardiotoxicity risk.

Most predictors included in the 6 prognostic models of this review were clinical variables. Incorporating a greater number of variables such as biomarkers (cardiac troponin and natriuretic peptides) or genetic information (microRNAs, proteomics and metabolomics) is another potential way to improve the prediction tool.^31^ Moreover, because in different patients, the same biomarker level could have a different meaning depending on whether it is stable or rising, repeated measurements of multiple biomarkers reflecting different pathophysiological pathways may convey incremental prognostic value to single or even repeated biomarker measurements of established biomarkers.^32^ The cumulative dose of anthracyclines has been proven to be an important factor associated with cardiotoxicity in patients receiving anthracycline-containing therapy.^33,34^ In our study, models that included both the cumulative dose of anthracyclines and several comorbidities showed a better predictive performance than models that did not include these variables, except for the study by Goel et al,^19^ that had excellent discrimination using only baseline and change in LVEF. This suggests that the cumulative dose of anthracyclines could play a large role in the risk of cardiotoxicity in patients with breast cancer.

Model overfitting and the resulting optimism are important concerns in prediction models. We can define overfitting specific to prediction models as fitting a statistical model with too many degrees of freedom in the modeling process.^24^ Although 5 studies conducted internal validations, only 2 studies^16,21^ applied internal validation properly by using bootstrapping techniques to overcome overfitting. In addition, although the demonstration of the performance of a model in an independent population is a necessary step before recommending its widespread use, only one of these models was externally validated.^35^ Inappropriate EPV for sample size can cause high risks of overfitting and biased predictions.^36^ EPV above 20 is recommended as the minimum sample size for model development^37^ and validation samples should contain at least 100 individuals who develop the outcome of interest.^38^ In this review, the sample size of 3 development models (EPV below 20) and the external model validation (less than 100 events) was not appropriate.

### Applicability of Findings to Clinical Practice

While the key messages were consistent, 4 included studies were at high overall ROB. One predictive model was only validated once, and we did not consider there to be enough information to assess its suitability in different situations. This review reveals that there is insufficient evidence to provide personalized risk prediction of cardiotoxicity related to breast cancer treatment in clinical practice.

### Strengths and Limitations

To the best of our knowledge, this is the first systematic review to focus on prediction models of cardiotoxicity in patients with breast cancer, including a thorough evaluation of the ROB and reporting quality. A main strength of this study is the adoption of the PROBAST and TRIPOD guidelines as benchmarks.

However, we acknowledge that there were several limitations to this systematic review. We only included studies published in English and did not search gray literature. However, it is unlikely that any missed studies would have significantly changed our main findings. Another weakness was that we were unable to perform our planned meta-analysis due to the lack of eligible studies.

## Conclusions

This review identified 6 prognostic models developed to predict risk of cardiotoxicity following treatment in women with breast cancer. The prediction models were designed in diverse clinical settings and populations, and with a range of included predictors. We are not yet able to accurately predict cardiotoxicity outcomes for a given patient with breast cancer based on their clinical parameters. Most of the models identified in this review fell short in addressing bias due to methodological weaknesses. To improve the overall quality in future predictive models, researchers should adhere to best practices in the development process. Investigators should also be aware of the potential benefits and weaknesses of various methods for model creation and internal validation. More external validation studies are needed to assess the performance and clinical applicability of existing models before they can successfully be used in clinical practice.

## Notes

Supplement 1. **eTable 1.** PRISMA Statement, Information Flow Through the Systematic Search Process **eTable 2.** PRISMA Statement for Abstract Checklist **eAppendix 1.** Search Strategy Conducted on September 22, 2021 **eAppendix 2.** Data Collection Questionnaire **eAppendix 3.** PROBAST Questionnaire **eAppendix 4.** TRIPOD Checklist **eTable 3.** Papers Excluded on Full-text **eTable 4.** Overview of Calculation per Included Model **eTable 5.** Regression Models **eTable 6.** PROBAST Quality Analysis Results **eFigure 1.** PROBAST Quality Analysis Results of 07 Studies (06 Model Development Studies and 01 Model Validation Studies) **eFigure 2.** TRIPOD Checklist

Supplement 2.
Data Sharing Statement
