# Supplementary material for: Risk Prediction Models for Cardiotoxicity of Chemotherapy Among Patients With Breast Cancer: A Systematic Review
Source: JAMA Netw Open. 2023 Feb 23;6(2):e230569. doi: 10.1001/jamanetworkopen.2023.0569 (PMC9951037; doi:10.1001/jamanetworkopen.2023.0569)
Supplement: Supplement 1. — eTable 1. PRISMA Statement, Information Flow Through the Systematic Search Process eTable 2. PRISMA Statement for Abstract Checklist eAppendix 1. Search Strategy Conducted on September 22, 2021 eAppendix 2. Data Collection Questionnaire eAppendix 3. PROBAST Questionnaire eAppendix 4. TRIPOD Checklist eTable 3. Papers Excluded on Full-text eTable 4. Overview of Calculation per Included Model eTable 5. Regression Models eTable 6. PROBAST Quality Analysis Results eFigure 1. PROBAST Quality Analysis Results of 07 Studies (06 Model Development Studies and 01 Model Validation Studies) eFigure 2. TRIPOD Checklist [file jamanetwopen-e230569-s001.pdf]

Supplemental Online Content

Kaboré EG, Macdonald C, Kaboré A, et al. Risk prediction models for cardiotoxicity of chemotherapy among patients with breast cancer: a systematic review. *JAMA Netw Open.* 2023;6(2):e230569. doi:10.1001/jamanetworkopen.2023.0569

eAppendix 1. Search Strategy Conducted on September 22, 2021

eAppendix 2. Data Collection Questionnaire

eAppendix 3. PROBAST Questionnaire

eAppendix 4. TRIPOD Checklist

eTable 1. Papers Excluded on Full-text

eTable 2. Overview of Calculation per Included Model

eTable 3. Regression Models

eTable 4. PROBAST Quality Analysis Results

eFigure 1. PROBAST Quality Analysis Results of 07 Studies (06 Model Development Studies and 01 Model Validation Studies)

eFigure 2. TRIPOD Checklist

This supplemental material has been provided by the authors to give readers additional information about their work.

© 2023 Kaboré EG et al. *JAMA Network Open.*

eAppendix 1. Search strategy conducted on September 22, 2021

Supplementary table 1: Search strategy used in Embase
(('breast tumor'/exp OR 'breast tumor':ab,ti OR (('malignant neoplasm'/exp OR 'cancer':ab,ti OR 'carcinoma’:ab,ti OR 'tumor’:ab,ti) AND ('breast':ab,ti OR 'breasts’:ab,ti OR ‘mamma*':ab,ti)))<br>AND<br>(‘prediction':ab,ti OR ‘predict’ OR ‘predictive’ OR ‘probability':ab,ti OR ‘prognosis':ab,ti OR ‘prognostic':ab,ti OR ‘prognostication':ab,ti OR ‘score':ab,ti OR ‘scores':ab,ti OR ‘independent*':ab,ti)<br>AND<br>(‘model':ab,ti OR ‘model*':ab,ti OR ‘regression':ab,ti OR ‘equation':ab,ti OR ‘equations':ab,ti OR ‘score':ab,ti OR ‘scores':ab,ti OR ‘probability':ab,ti OR ‘prognosis':ab,ti OR ‘prognos*':ab,ti)<br>AND<br>(‘heart failure':ab,ti OR ‘cardiotoxicity':ab,ti OR ‘cardiotoxic*':ab,ti OR (‘cardiac':ab,ti AND ‘toxicit*':ab,ti))<br>AND<br>(‘prediction':ab,ti OR ‘predict':ab,ti OR ‘predictive':ab,ti OR ‘predicting':ab,ti OR ‘validation':ab,ti OR ‘validity':ab,ti OR ‘validated':ab,ti OR ‘cross-validation':ab,ti OR ‘selection':ab,ti OR ‘calibration':ab,ti OR ‘discrimination':ab,ti OR ‘discriminates':ab,ti OR ‘ROC':ab,ti))

© 2023 Kaboré EG et al. *JAMA Network Open.*

eAppendix 2. Data collection questionnaire

Supplementary table 3. Data collection questionnaire	
Study characteristics	
General information	Model and type of study
	Secondary citations
	Language of publication
	Study design
	Follow-up time
Participants	Number of included persons in the cohort
	Setting
	Recruitment period
	Age (in years)
	Sex
	Stages of disease
	Treatment
	Inclusion criteria
	Exclusion criteria
Predictors	List of predictors in final model (including cut-points for dichotomised factors)
	Timing of predictor measurement
Outcome(s)	Primary outcome in study
	Additional outcome(s)
	Outcome in model development
Missing data	Participants with any missing data?
	If yes, how was missing data handled?
Analysis	Number of participants and number of events (specific time points where reported)
	Predictor selection method
	Statistical method
	Simplification of model?
	Performance measures reported?
	Creation of risk groups?
PROBAST: Applicability	Domain 1: Participant selection
	Domain 2: Predictors
	Domain 3: Outcome
Notes	Funding and conflict of interest
	Other comments

**Item**	**Authors' judgement**	**Support for judgement**
Domain 1: Participant selection		

© 2023 Kaboré EG et al. *JAMA Network Open.*

Domain 2: Predictors		
Domain 3: Outcome		
Domain 4: Analysis		
Overall judgement		

© 2023 Kaboré EG et al. *JAMA Network Open.*

eAppendix 3. PROBAST questionnaire

**Supplementary Material III.** PROBAST questionnaire
**DOMAIN 1: Participants**<br>*A. Risk of Bias*<br><br>Describe the sources of data and criteria for participant selection:<br>1.1 Were appropriate data sources used, e.g. cohort, RCT or nested case-control study data?<br>1.2 Were all inclusions and exclusions of participants appropriate?<br>Risk of bias introduced by selection of participants RISK: (low/ high/ unclear)<br>Rationale of bias rating:<br>*B. Applicability*<br><br>Describe included participants, setting and dates:<br>Concern that the included participants and setting do not match the review question:<br>CONCERN: (low/ high/ unclear)<br>Rationale of applicability rating:
**DOMAIN 2: Predictors**<br>*A. Risk of Bias*<br><br>List and describe predictors included in the final model, e.g. definition and timing of assessment:<br>2.1 Were predictors defined and assessed in a similar way for all participants?<br>2.2 Were predictor assessments made without knowledge of outcome data?<br>2.3 Are all predictors available at the time the model is intended to be used?<br>Risk of bias introduced by predictors or their assessment RISK: (low/ high/ unclear)<br>Rationale of bias rating:<br>*B. Applicability*<br><br>Concern that the definition, assessment or timing of predictors in the model do not match the review question:<br>CONCERN: (low/ high/ unclear)<br>Rationale of applicability rating:
**DOMAIN 3: Outcome**<br>*A. Risk of Bias*<br><br>Describe the outcome, how it was defined and determined, and the time interval between predictor assessment and outcome determination:<br>3.1 Was the outcome determined appropriately?<br>3.2 Was a pre-specified or standard outcome definition used?<br>3.3 Were predictors excluded from the outcome definition?<br>3.4 Was the outcome defined and determined in a similar way for all participants?<br>3.5 Was the outcome determined without knowledge of predictor information?<br>3.6 Was the time interval between predictor assessment and outcome determination appropriate?<br>Risk of bias introduced by the outcome or its determination RISK: (low/ high/ unclear)<br>Rationale of bias rating:<br>*B. Applicability*<br><br>At what time point was the outcome determined:<br>If a composite outcome was used, describe the relative frequency/distribution of each contributing outcome:<br>Concern that the outcome, its definition, timing or determination do not match the review question:

CONCERN: (low/ high/ unclear)
Rationale of applicability rating:

DOMAIN 4: Analysis
Risk of Bias

Describe numbers of participants, number of candidate predictors, outcome events and events per candidate predictor:

Describe how the model was developed (for example in regards to modelling technique (e.g. survival or logistic modelling), predictor selection, and risk group definition):

Describe whether and how the model was validated, either internally (e.g. bootstrapping, cross validation, random split sample) or externally (e.g. temporal validation, geographical validation, different setting, different type of participants):

Describe the performance measures of the model, e.g. (re)calibration, discrimination, (re)classification, net benefit, and whether they were adjusted for optimism:

Describe any participants who were excluded from the analysis:

Describe missing data on predictors and outcomes as well as methods used for missing data:

4.1 Were there a reasonable number of participants with the outcome?
4.2 Were continuous and categorical predictors handled appropriately?
4.3 Were all enrolled participants included in the analysis?
4.4 Were participants with missing data handled appropriately?
4.5 Was selection of predictors based on univariable analysis avoided?
4.6 Were complexities in the data (e.g. censoring, competing risks, sampling of controls) accounted for appropriately?
4.7 Were relevant model performance measures evaluated appropriately?
4.8 Were model overfitting and optimism in model performance accounted for?
4.9 Do predictors and their assigned weights in the final model correspond to the results from multivariable analysis?
Risk of bias introduced by the analysis RISK: (low/ high/ unclear)
Rationale of bias rating:

© 2023 Kaboré EG et al. *JAMA Network Open.*

eAppendix 4. TRIPOD Checklist

Section/Topic	Item	Development or Validation?	Checklist Item
**Title and abstract**			
Title	1	D;V	Identify the study as developing and/or validating a multivariable prediction model, the target population, and the outcome to be predicted.
Abstract	2	D;V	Provide a summary of objectives, study design, setting, participants, sample size, predictors, outcome, statistical analysis, results, and conclusions.
**Introduction**			
Background and objectives	3a	D;V	Explain the medical context (including whether diagnostic or prognostic) and rationale for developing or validating the multivariable prediction model, including references to existing models.
	3b	D;V	Specify the objectives, including whether the study describes the development or validation of the model, or both.
**Methods**			
Source of data	4a	D;V	Describe the study design or source of data (e.g., randomized trial, cohort, or registry data), separately for the development and validation datasets, if applicable.
	4b	D;V	Specify the key study dates, including start of accrual; end of accrual; and, if applicable, end of follow-up.
Participants	5a	D;V	Specify key elements of the study setting (e.g., primary care, secondary care, general population) including number and location of centres.
	5b	D;V	Describe eligibility criteria for participants.
	5c	D;V	Give details of treatments received, if relevant.
Outcome	6a	D;V	Clearly define the outcome that is predicted by the prediction model, including how and when assessed.
	6b	D;V	Report any actions to blind assessment of the outcome to be predicted.
Predictors	7a	D;V	Clearly define all predictors used in developing the multivariable prediction model, including how and when they were measured.
	7b	D;V	Report any actions to blind assessment of predictors for the outcome and other predictors.
Sample size	8	D;V	Explain how the study size was arrived at.
Missing data	9	D;V	Describe how missing data were handled (e.g., complete-case analysis, single imputation, multiple imputation) with details of any imputation method.
Statistical analysis methods	10a	D	Describe how predictors were handled in the analyses.
	10b	D	Specify type of model, all model-building procedures (including any predictor selection), and method for internal validation.
	10c	V	For validation, describe how the predictions were calculated.
	10d	D;V	Specify all measures used to assess model performance and, if relevant, to compare multiple models.
	10e	V	Describe any model updating (e.g., recalibration) arising from the validation, if done.
Risk groups	11	D;V	Provide details on how risk groups were created, if done.
Development vs. validation	12	V	For validation, identify any differences from the development data in setting, eligibility criteria, outcome, and predictors.
Results			
Participants	13a	D;V	Describe the flow of participants through the study, including the number of participants with and without the outcome and, if applicable, a summary of the follow-up time. A diagram may be helpful.
	13b	D;V	Describe the characteristics of the participants (basic demographics, clinical features, available predictors), including the number of participants with missing data for predictors and outcome.
	13c	V	For validation, show a comparison with the development data of the distribution of important variables (demographics, predictors, and outcome).
Model development	14a	D	Specify the number of participants and outcome events in each analysis.
	14b	D	If done, report the unadjusted association between each candidate predictor and outcome.
Model specification	15a	D	Present the full prediction model to allow predictions for individuals (i.e., all regression coefficients, and model intercept or baseline survival at a given time point).
	15b	D	Explain how to use the prediction model.
Model performance	16	D;V	Report performance measures (with CIs) for the prediction model.
Model updating	17	V	If done, report the results from any model updating (i.e., model specification, model performance).
**Discussion**			
Limitations	18	D;V	Discuss any limitations of the study (such as nonrepresentative sample, few events per predictor, missing data).
Interpretations	19a	V	For validation, discuss the results with reference to performance in the development data, and any other validation data.
	19b	D;V	Give an overall interpretation of the results, considering objectives, limitations, results from similar studies, and other relevant evidence.
Implications	20	D;V	Discuss the potential clinical use of the model and implications for future research.
**Other information**			
Supplementary information	21	D;V	Provide information about the availability of supplementary resources, such as study protocol, Web calculator, and datasets.
Funding	22	D;V	Give the source of funding and the role of the funders for the present study.

© 2023 Kaboré EG et al. *JAMA Network Open.*

eTable 1. Papers excluded on full-text

Supplementary table 2. Papers excluded on full-text	
Paper ID	Reasons for exclusion
Abdel-Qadir (2019)<br>Abdel-Qadir H, Thavendiranathan P, Austin PC, Lee DS, Amir E, Tu JV, et al. Development and validation of a multivariable prediction model for major adverse cardiovascular events after early stage breast cancer: a population-based cohort study. Eur Heart J. 2019;40(48):3913-20.	Data on cardiotoxic cancer therapies not included
Battisti (2021)<br>Battisti NML, Andres MS, Lee KA, Ramalingam S, Nash T, Mappouridou S, et al. Incidence of cardiotoxicity and validation of the Heart Failure Association-International Cardio-Oncology Society risk stratification tool in patients treated with trastuzumab for HER2-positive early breast cancer. Breast Cancer Res Treat. 2021;188(1):149-63.	Validation study of consensus score
Dranitsaris (2008)<br>Dranitsaris G, Rayson D, Vincent M, Chang J, Gelmon K, Sandor D, et al. The development of a predictive model to estimate cardiotoxic risk for patients with metastatic breast cancer receiving anthracyclines. Breast Cancer Res Treat. 2008;107(3):443-50.	Only metastatic breast cancer patients
Gaasch (2020)<br>Gaasch A, Schönecker S, Simonetto C, Eidemüller M, Pazos M, Reitz D, et al. Heart sparing radiotherapy in breast cancer: the importance of baseline cardiac risks. Radiat Oncol. 2020;15(1):117.	prognostic factor study
Ky (2014)<br>Ky B, Putt M, Sawaya H, French B, Januzzi JL, Jr., Sebag IA, et al. Early increases in multiple biomarkers predict subsequent cardiotoxicity in patients with breast cancer treated with doxorubicin, taxanes, and trastuzumab. J Am Coll Cardiol. 2014;63(8):809-16.	prognostic factor study
Law (2017)<br>Law W, Johnson C, Rushton M, Dent S. The Framingham risk score underestimates the risk of cardiovascular events in the HER2-positive breast cancer population. Curr Oncol. 2017;24(5):e348-e53.	prognostic factor study
Nakano (2019)<br>Nakano MH, Udagawa C, Shimo A, Kojima Y, Yoshie R, Zaha H, et al. A Genome-Wide Association Study Identifies Five Novel Genetic Markers for Trastuzumab-Induced Cardiotoxicity in Japanese Population. Biol Pharm Bull. 2019;42(12):2045-53.	Genetic analyses/ongoing study
Narayan (2019)<br>Narayan HK, French B, Khan AM, Plappert T, Hyman D, Bajulaiye A, et al. Noninvasive Measures of Ventricular-Arterial Coupling and Circumferential Strain Predict Cancer Therapeutics-Related Cardiac Dysfunction. JACC Cardiovasc Imaging. 2016;9(10):1131-41.	prognostic factor study
Öztürk (2021)<br>Öztürk C, Validyev D, Becher UM, Weber M, Nickenig G, Tiyerili V. A novel scoring system to estimate chemotherapy-induced myocardial toxicity: Risk assessment prior to non-anthracycline chemotherapy regimens. IJC Heart & Vasculature. 2021;33:100751	Participant eligibility criteria not match:<br>Male and female participants;<br>Hematological, gastrointestinal, lung and breast cancer
Rushton (2017)	Not a model

© 2023 Kaboré EG et al. *JAMA Network Open.*

Rushton M, Johnson C, Dent S. Trastuzumab-induced cardiotoxicity: testing a clinical risk score in a real-world cardio-oncology population. Curr Oncol. 2017;24(3):176-80.	

© 2023 Kaboré EG et al. *JAMA Network Open.*

eTable 2. Overview of calculation per included model

Supplementary Table 4. Overview of calculation per included model					
Model	Developed for patient treated with:	Outcome	Variable categories	Points	Probability of outcome
Ezaz et al (1), 2014;	ANTH chemotherapy: 35.9%; non-ANTH chemotherapy: 47.7%; No identified chemotherapy: 16.4%	3-Year Risk of HF/CM	age 67-74	0	0-3=14.5%<br>4-5=26.2%<br>≥6=42.9%
			age 75-79 years	1	
			age 80-94 years	2	
			anthracycline chemotherapy	2	
			non-anthracycline chemotherapy	2	
			no identified chemotherapy	0	
			coronary artery disease	2	
			atrial fibrillation/flutter	2	
			diabetes mellitus	1	
			hypertension	1	
			Renal failure	2	
			Total score	0-12	
Fogarassy et al (2), 2019;	100% epirubicin; 20% with targeted therapies	3–10-year Risk of HF	Age 40-49 years	3	-1-7=2.1%<br>8-9=5%<br>10-12=10.3%<br>13-18=22.1%<br>19-26=31.7%
			age 50-59 years	4	
			age 60-69 years	7	
			age ≥70 years,	10	
			Diabetes,	2	
			hypertension,	1	
			CAD without myocardial or revascularization	1	
			CAD with myocardial or revascularization	3	
			previous stroke	2	
			Stage regional invasion	1	
			Stage distant metastasis	4	
			Epirubicin dose 451-540 mg/m²	0	
			Epirubicin dose 541-709mg/m²	2	
			Epirubicin dose>709mg/m²,	3	
			Docetaxel dose ≤510 mg/m²	1	
			docetaxel dose > 510mg/m²	2	
			Capecitabine	4	
			Gemcitabine	3	
			bevacizumab	3	
			ACEi/ARB	-1	
			Total score	-1-37	
CHEMO-RADIAT;<br>Kim et al (3), 2021;	ANTH:70%; TRZ: 11.9%	1-3-7-year Risk of MACE	Congestive HF	2	0–2=0.08 per 100 person-years<br>3–5=?<br>≥6 =?
			Hypertension,	1	
			Elderly (age ≥60),	1	
			Myocardial infarction/peripheral artery occlusive disease,	2	
			Obesity	1	
			Renal failure (estimated glomerular filtration	1	

© 2023 Kaboré EG et al. *JAMA Network Open.*

			rate <60 mL/min per 1.73 m²)		
			Abnormal lipid profile (dyslipidemia)	1	
			Diabetes mellitus	1	
			Irradiation to left breast with ≥30 Gy dose	1	
			Anthracycline dose (doxorubicin equivalent dose: for daunorubicin multiply by 0.5, for epirubicin multiply by 0.5, for idarubicin multiply by 2)	1 per each 100 mg/ m2 (rounded to nearest integer)	
			TIA/stroke	2	
			Total score	0-15	

© 2023 Kaboré EG et al. *JAMA Network Open.*

eTable 3. Regression models

Supplementary Table IV. Regression models				
Model	Developed for patient treated with:	Outcome	Equation	Probability
Low risk TRC; Goel et al (4), 2019;	ANTH-based chemotherapy followed by taxane chemotherapy given with TRZ, followed by TRZ alone	1-year risk of trastuzumab-related cardiotoxicity	3 × (baseline LVEF) - 4.3 × (LVEF change)	>201%=1.2%
CRS; Romond et al (5), 2012;	Arm 1: doxorubicin and cyclophosphamide followed by paclitaxel<br>Arm 2: same chemotherapy plus TRZ	5-year risk of cardiac death or congestive HF	[(7.0 + (0.04 × Age in years) - (0.1 × Baseline percent LVEF)) × 100]/4.76	CRS calculation can then be plotted on a Graph to obtain the estimated predicted probability
Upshaw et al (6), 2019; Development model	doxorubicin and cyclophosphamide followed by paclitaxel	1-year risk of cardiotoxicity	-2.64+0.04 × Age + 0.05 × Body Mass Index -0.26 × Hypertension – 0.06 × Baseline LVEF	Transformation to probability from log odds $P = \frac{1}{1+e^{-\log(odds)}}$

© 2023 Kaboré EG et al. *JAMA Network Open.*

eTable 4. PROBAST quality analysis Results

Supplement table 4. PROBAST quality analysis Results*											
**Study**	**ROB**					**Applicability**				**Overall**	
	**Participants**	**Predictors**	**Outcome**	**Analysis**		**Participants**	**Predictors**	**Outcome**		**ROB**	**Applicability**
Ezaz, 2014	+	+	+	-		+	+	+		-	+
Fogarassy, 2019	+	+	+	+		+	+	+		+	+
Goel, 2019	+	+	+	-		+	+	+		-	+
Kim, 2021	+	+	+	-		+	+	+		-	+
Milks, 2018	?	+	+	-		?	+	+		-	?
Romond, 2012	+	+	+	-		+	+	+		-	+
Upshaw, 2019	+	+	+	?		+	+	+		?	+

PROBAST = Prediction model Risk Of Bias ASsessment Tool; ROB = risk of bias.
* + indicates low ROB/low concern regarding applicability; − indicates high ROB/high concern regarding applicability; and ? indicates unclear ROB/unclear concern regarding applicability

© 2023 Kaboré EG et al. *JAMA Network Open.*

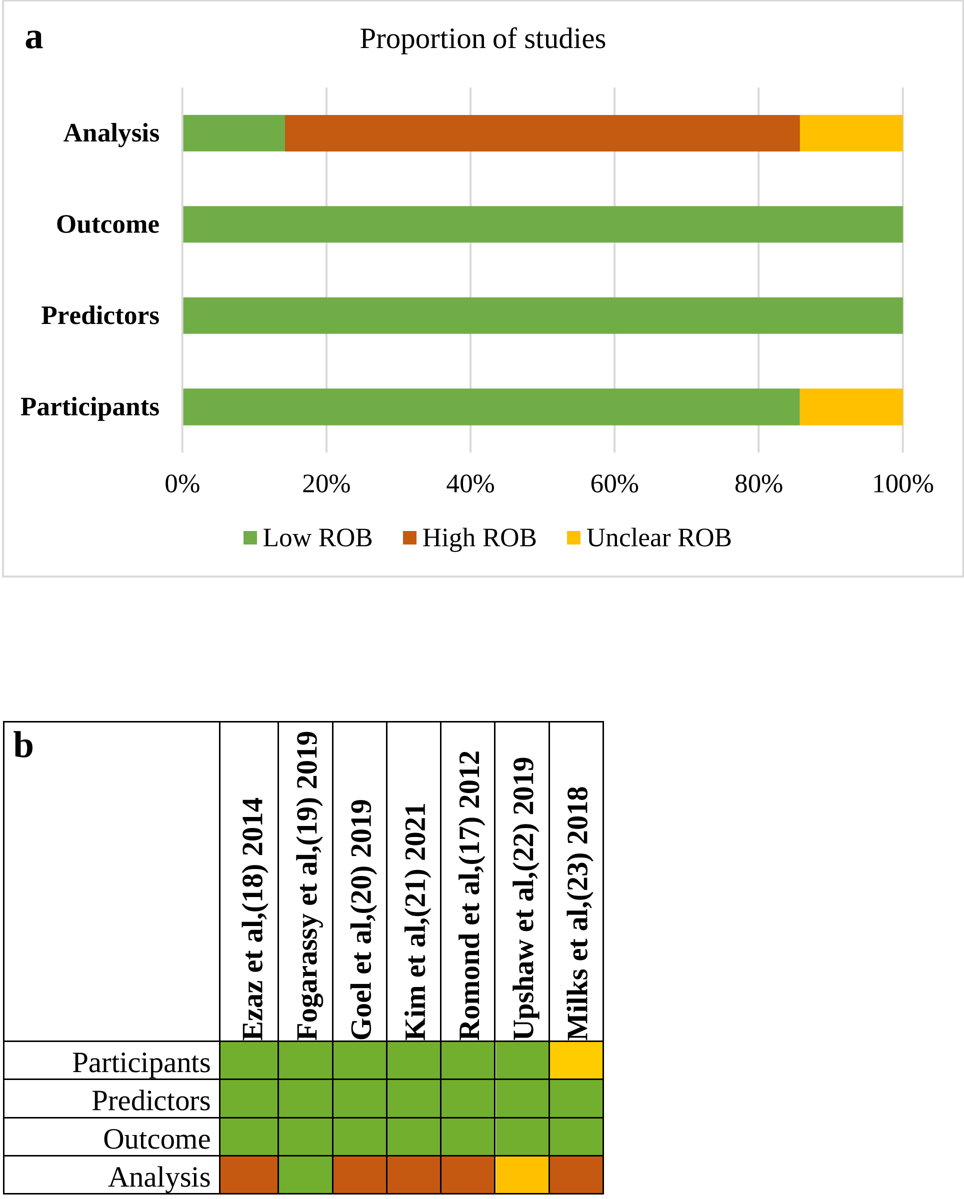

eFigure 1. PROBAST quality analysis results of 07 studies (06 model development studies and 01 model validation studies). **a)** Overall rate of risk of bias (ROB): low, green; high, orange; unclear, yellow. **b)** Risk of bias in each study.

© 2023 Kaboré EG et al. *JAMA Network Open.*

	2 Abstract-Summary	3a Introduction	3b The objectives	4a Methods-Study design	5a The study setting	5b Elligibility criteria	5c Treatment, if relevant	6a Outcome definition	7a Predictors definition	8 Study size	10a (D) Handling predictors	10b (D) Model development	13b Participants characteristics	14a (D) Number of outcome	15a (D) Full model presentation	15b (D) How to use the model	18 Discussion-study limitations	19b Overall interpretation	20 Potential clinical use	21 Supplementary information	22 Funding	1 Title	4b The key study dates	9 Handling missing data	11 Risk groups	13a Flow of participants selection	10d Assessing model performance	16 Performance measures	14b (D) unajusted association, if done	6b Blind assessment of outcome	7b Blind assessment of predictors	**Total items reported**
Kim et al,(21) 2021	Reported	Reported	Reported	Reported	Reported	Reported	Reported	Reported	Reported	Reported	Reported	Reported	Reported	Reported	Reported	Reported	Reported	Reported	Reported	Reported	Reported	Reported	Reported	Reported	Reported	Reported	Reported	Reported	Not applicable	Not applicable	Not applicable	**28**
Romond et al,(17) 2012	Reported	Reported	Reported	Reported	Reported	Reported	Reported	Reported	Reported	Reported	Reported	Reported	Reported	Reported	Reported	Reported	Reported	Reported	Reported	Reported	Reported	Reported	Reported	Reported	Not applicable	Reported	Reported	Reported	Not reported	Not reported	Not reported	**27**
Fogarassy et al,(19) 2019	Reported	Reported	Reported	Reported	Reported	Reported	Reported	Reported	Reported	Reported	Reported	Reported	Reported	Reported	Reported	Reported	Reported	Reported	Reported	Reported	Reported	Reported	Reported	Not reported	Reported	Not reported	Reported	Reported	Not applicable	Not applicable	Not applicable	**26**
Upshaw et al,(22) 2019	Reported	Reported	Reported	Reported	Reported	Reported	Reported	Reported	Reported	Reported	Reported	Reported	Reported	Reported	Reported	Reported	Reported	Reported	Reported	Reported	Reported	Reported	Reported	Reported	Reported	Not reported	Not reported	Not reported	Not applicable	Not reported	Not reported	**25**
Ezaz et al,(18) 2014	Reported	Reported	Reported	Reported	Reported	Reported	Reported	Reported	Reported	Reported	Reported	Reported	Reported	Reported	Reported	Reported	Reported	Reported	Reported	Reported	Reported	Reported	Reported	Reported	Reported	Not reported	Not reported	Not reported	Not reported	Not reported	Not reported	**25**
Goel et al,(20) 2019	Reported	Reported	Reported	Reported	Reported	Reported	Reported	Reported	Reported	Reported	Reported	Reported	Reported	Reported	Reported	Reported	Reported	Reported	Reported	Reported	Reported	Not reported	Not reported	Reported	Reported	Reported	Not reported	Not reported	Reported	Not reported	Not reported	**25**
**Total studies reported**	**6**	**6**	**6**	**6**	**6**	**6**	**6**	**6**	**6**	**6**	**6**	**6**	**6**	**6**	**6**	**6**	**6**	**6**	**6**	**6**	**6**	**5**	**5**	**5**	**5**	**3**	**3**	**3**	**1**	**0**	**0**	

Reported Not reported Not applicable

eFigure 2. TRIPOD checklist. Completeness of TRIPOD checklist for six model development studies. Right columns are the least-reported items, and the bottom studies represent the least-reported studies.

© 2023 Kaboré EG et al. *JAMA Network Open.*
