# Supplementary material for: Risk Prediction Models for Cardiotoxicity of Chemotherapy Among Patients With Breast Cancer: A Systematic Review
Source: JAMA Netw Open. 2023 Feb 23;6(2):e230569. doi: 10.1001/jamanetworkopen.2023.0569 (PMC9951037; doi:10.1001/jamanetworkopen.2023.0569)
Supplement: Supplement 2. — Data Sharing Statement [file jamanetwopen-e230569-s002.pdf]

Data Sharing Statement

Kaboré EG, Macdonald C, Kaboré A, et al. Risk prediction models for cardiotoxicity of chemotherapy among patients with breast cancer. *JAMA Netw Open.* Published February 23, 2023. doi:10.1001/jamanetworkopen.2023.0569

Data
Data available: Yes
Data types: Data (not involving human participants)
How to access data: kabelisee@yahoo.fr
When available: With publication

Supporting Documents
Document types: Statistical/analytic code
How to access documents: kabelisee@yahoo.fr
When available: With publication

Additional Information
Who can access the data: kabelisee@yahoo.fr
Types of analyses: Data extraction
Mechanisms of data availability: with investigator support
